# Highly Compressible and Sensitive Flexible Piezoresistive Pressure Sensor Based on MWCNTs/Ti_3_C_2_T_x_ MXene @ Melamine Foam for Human Gesture Monitoring and Recognition

**DOI:** 10.3390/nano12132225

**Published:** 2022-06-29

**Authors:** Yue Su, Kainan Ma, Xurui Mao, Ming Liu, Xu Zhang

**Affiliations:** Institute of Semiconductors, Chinese Academy of Sciences, Beijing 100864, China; yuesu@semi.ac.cn (Y.S.); makainan@semi.ac.cn (K.M.); maoxurui@semi.ac.cn (X.M.)

**Keywords:** foam-shaped piezoresistive pressure sensor, MWCNTs, Ti_3_C_2_T_x_ MXene, porous skeleton, gesture recognition

## Abstract

Flexible sensing devices provide a convenient and effective solution for real-time human motion monitoring, but achieving efficient and low-cost assembly of pressure sensors with high performance remains a considerable challenge. Herein, a highly compressible and sensitive flexible foam-shaped piezoresistive pressure sensor was prepared by sequential fixing multiwalled carbon nanotubes and ${\mathrm{Ti}}_{3}C_{2}T_{x}$ MXene on the skeleton of melamine foam. Due to the porous skeleton of the melamine foam and the extraordinary electrical properties of the conductive fillers, the obtained MWCNTs/${\mathrm{Ti}}_{3}C_{2}T_{x}$ MXene @ melamine foam device features high sensitivity of 0.339 kPa${}^{-1}$, a wide working range up to 180 kPa, a desirable response time and excellent cyclic stability. The sensing mechanism of the composite foam device is attributed to the change in the conductive pathways between adjacent porous skeletons. The proposed sensor can be used successfully to monitor human gestures in real-time, such as finger bending and tilting, scrolling the mouse and stretching fingers. By combining with the decision tree algorithm, the sensor can unambiguously classify different Arabic numeral gestures with an average recognition accuracy of 98.9%. Therefore, our fabricated foam-shaped sensor may have great potential as next-generation wearable electronics to accurately acquire and recognize human gesture signals in various practical applications.

## 1. Introduction

With the rapid advancement of Artificial Intelligence, human gesture recognition has received extensive research attention due to its applications in sports rehabilitation, human–machine interfaces and many other fields [1,2]. Conventional sensing devices, such as optical fibers and inertial measurement units [3,4,5], have been developed to acquire and recognize gesture signals; however, the measurement accuracy suffers due to poor conformability to human skin. Recently, flexible pressure sensors that can be arbitrarily twisted and attached to non-planar human skin surfaces have been designed and applied in the field of hand gesture recognition.

These flexible devices are mainly classified into four categories, namely piezoelectricity [6,7], piezoresistivity [8,9], capacitance [10] and triboelectricity [11], according to the various transduction mechanisms. Among them, the flexible resistive-type sensors that can convert mechanical force into readable electrical resistance variation have been commonly researched due to their advantages of low cost, convenient manufacturing techniques and easy signal acquisition [12,13]. Two-dimensional microstructures of different designs, such as micro-pyramid [14], pillars [15], hemispheres [16] and molded bionic structures [17,18], have been adopted for improving the sensing performance of piezoresistive devices.

Although the sensing properties, such as the response time, working range, sensitivity and mechanical compliance, can be significantly improved, challenges remain to achieve high sensitivity over a wide sensing range. Recently, three-dimensional microstructures, composed of conductive materials (e.g., graphene [19,20], carbon nanotubes [21,22] and metal nanowires [23,24]) combined with porous framework polymers, have been extensively studied and become promising candidates for the fabrication of flexible piezoresistive sensors with a broad sensitive range.

These three-dimensional microstructures are synthesized and prepared by different chemical or physical methods, including freeze-drying, chemical vapor deposition (CVD), dip coating, and sputtering [25]. For example, the flexible CuRGOMF (copper nanowires/reduced graphene oxide/melamine foam) sensor was acquired via coating RGO (reduced graphene oxide) and in situ growing of CuNWs (copper nanowires) on the skeleton of MF (melamine foam) [26]. The as-prepared sensor of CuRGOMF features high sensitivities of 0.011 kPa${}^{-1}$ and 0.088 kPa${}^{-1}$ over wide working ranges of 0–1.5 kPa and 1.5–10 kPa, respectively, and can be successfully used for the monitoring of human motions.

The flexible graphene-PDMS @ sponge piezoresistive sensor, with a high sensitivity of 0.075 kPa${}^{-1}$ and a wide responding range up to 50 kPa, was prepared via stabilizing graphene conductive nanomaterials on a sponge skeleton using PDMS [25]. Despite the remarkable progress in 3D foam sensor research, there are still great challenges to realize the fabrication of higher-performance wearable foam sensing devices in an inexpensive, easy-to-operate and large-scale fabrication mode.

This paper proposes a compression- and pressure-sensitive foam-shaped piezoresistive pressure sensor by combining a simple dip-coating method to overcome the challenging problems mentioned above. The as-prepared foam sensing device comprises two-dimensional transition metal carbides, nitrides, carbonitrides (MXenes) with excellent metallic conductivity, multiwalled carbon nanotubes (MWCNTs) and melamine foam with high porosity of over 99%. Due to the rational selection of electrical conductive fillers and porous skeleton structure, the MWCNTs/${\mathrm{Ti}}_{3}C_{2}T_{x}$ MXene @ melamine foam sensor illustrates high compression sensitivity, broad sensing range, short response time and excellent stability.

The application research reveals that the obtained foam-shaped device can be used successfully to monitor various human gestures by sensing the bending and tilting states of the finger joints during specific movements. As one of the most influential machine learning algorithms for data classification, decision trees have been applied to many subject areas because they are efficient and easy to explain [27,28]. We further utilize a decision tree model to recognize a large amount of the acquired resistance data obtained by variations of ten typical Arabic numeral gestures, and the experimental results show that the combination of the proposed sensor and machine-learning method could classify the ten Arabic numeral gestures with an average recognition accuracy of 98.9%. The developed foam-shaped sensor has the promising potential to take on recognition tasks in sophisticated hand movement applications.

## 2. Experiments Details

**Figure 1:**
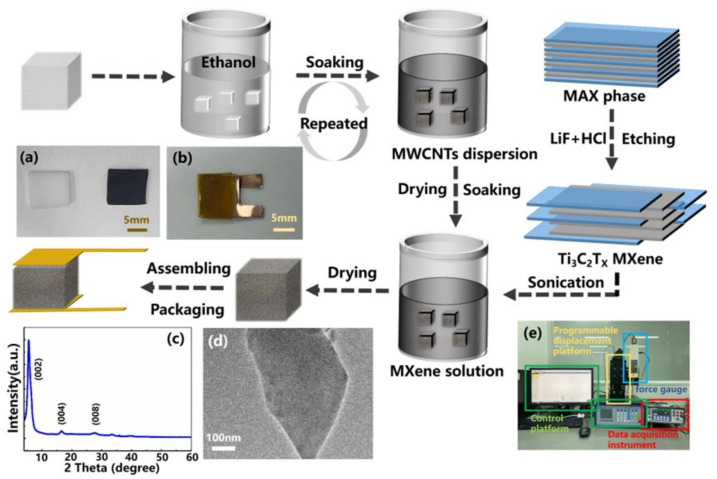
The manufacturing process of the MWCNTs/${\mathrm{Ti}}_{3}C_{2}T_{x}$ MXene @ melamine foam sensor. (**a**) Photos of the original melamine foam and the as-prepared MWCNTs/${\mathrm{Ti}}_{3}C_{2}T_{x}$ MXene @ melamine foam. (**b**) Photograph of the fabricated foam-shaped composite sensor taken by a digital camera. (**c**) X-ray diffraction (XRD) patterns of the MXene nanosheet. (**d**) TEM image of the MXene nanosheet. (**e**) The home-made mechanical testing system and the data acquisition instrument.

Figure 1 illustrates the manufacturing process of the MWCNTs/${\mathrm{Ti}}_{3}C_{2}T_{x}$ MXene @ melamine foam sensor.

**Preparation of ${\mathrm{Ti}}_{3}C_{2}T_{x}$ aqueous dispersions**

First, an etching solution was prepared by dissolving 2 g of LiF in 40 mL of 9 M HCl with magnetic stirring. After the solution was sufficiently cooled to room temperature, 2 g of MAX phase precursor (${\mathrm{Ti}}_{3}{\mathrm{AlC}}_{2}$) powder was slowly added to the above reaction system within 10 min to etch the Al layer selectively. Then, this MXene suspension was repeatedly diluted with deionized water and centrifuged (3500 rpm) until the pH was about 5–6. Afterwards, the ${\mathrm{Ti}}_{3}C_{2}T_{x}$ precipitate obtained above was dispersed in a certain amount of deionized water and ultrasonically mechanically peeled off under inert gas for 1 h.

Finally, the colloidal suspension of 2D ${\mathrm{Ti}}_{3}C_{2}T_{x}$ nanosheets, the dark green supernatant, was collected after centrifugation at 3500 rpm for 1 h. In this experiment, the concentration of the ${\mathrm{Ti}}_{3}C_{2}T_{x}$ MXene suspension was further regulated to 5 mg/mL for later usage. The XRD patterns spectra shown in the inset (c) of Figure 1 reveals the exhaustion of ${\mathrm{Ti}}_{3}{\mathrm{AlC}}_{2}$ and confirms that the derived MXene is phase pure, on account of the strong diffraction peak (002) located at 6${}^{{}^{\circ}}$ and the removal of the (104) peak related to the Al element around 39${}^{{}^{\circ}}$.

Subsequently, the MXene (${\mathrm{Ti}}_{3}C_{2}T_{x}$) nanosheet solution is diluted and transferred onto copper grids with ultrathin holey carbon films for TEM characterization. From the transmission electron microscopy (TEM) image in the inset (d) of Figure 1, it can be found that the single-layer MXene nanosheet is transparent and ultrathin, exhibiting an excellent layered structure (the obtained thickness of the MXene nanosheet is 1.49 nm, indicating that the synthesized MXene is monolayer; not shown).

**Fabrication of the MWCNTs/${\mathrm{Ti}}_{3}C_{2}T_{x}$ MXene @ melamine foam sensor**

The commercially available melamine foam with pore density of 45PPI (pores per inch) was first cut into rectangular-shaped pieces of $10\times 10\times 1.5\;{\mathrm{mm}}^{3}$, then ultrasonically cleaned with acetone, ethanol and deionized water several times successively. After being dried in an oven at 50 °C for 2 h, the rectangular-shaped melamine sponges were soaked in 10 wt % MWCNTs aqueous dispersion for 10 min followed by drying in an oven at 70 °C for 1 h to eliminate the residual liquid. The MWCNTs @ melamine foam was obtained after repeating the above process twice.

Afterwards, this composite foam was dipped into a 5 mg/mL ${\mathrm{Ti}}_{3}C_{2}T_{x}$ MXene suspension for 10 min. After the ${\mathrm{Ti}}_{3}C_{2}T_{x}$ nanosheets were attached to the skeleton’s surface via Van der Waals force, the sample was dried at room temperature to obtain the MWCNTs/${\mathrm{Ti}}_{3}C_{2}T_{x}$ MXene @ melamine foam. The inset (a) of Figure 1 shows the photos of the pristine melamine foam and the MWCNTs/${\mathrm{Ti}}_{3}C_{2}T_{x}$ MXene @ melamine foam.

It can be seen that the color turns from the original white of melamine foam to the dark black of MWCNTs/${\mathrm{Ti}}_{3}C_{2}T_{x}$ MXene @ melamine foam, while the shape remained basically unchanged. The sandwich-structural MWCNTs/${\mathrm{Ti}}_{3}C_{2}T_{x}$ MXene @ melamine foam sensor was finally assembled by adhering two sheets of copper foils to the upper and lower surfaces of the composite foam with polyimide tape. The inset (b) in Figure 1 exhibits the photograph of the fabricated foam-shaped sensor with a sensing area size of 10×10 mm.

**Characterization**

The morphology, crystal structure and phase composition of MXene nanosheets were characterized by transmission electron microscopy (TEM, JEM-F200, JEOL) and an X-ray diffractometer (XRD, Rigaku D/MAX 2550). The thickness of the MXene nanosheet was investigated by an atomic force microscope (AFM, Shimadzu SPM9700).

A homemade mechanical testing system composed of a force gauge and a programmable displacement platform was constructed to apply controllable pressure, and the electrical resistance of the foam-shaped sensor was continuously recorded by using a data acquisition instrument (Keithley DAQ6510) as shown in the inset (e) of Figure 1. In this experiment, the base resistance of the foam-shaped composite sensor is 465 Ω. When the sensor resistance value is greater than 10 Ω, the test current used by the data acquisition instrument is 1 mA (±5%). When the resistance value is less than 10 Ω, the test current increases to 10 mA. The piezoresistivity of the composite foam sensor under compression was studied according to the electrical signal test results and the corresponding mechanical measurements.

## 3. Results and Discussion

**Figure 2:**
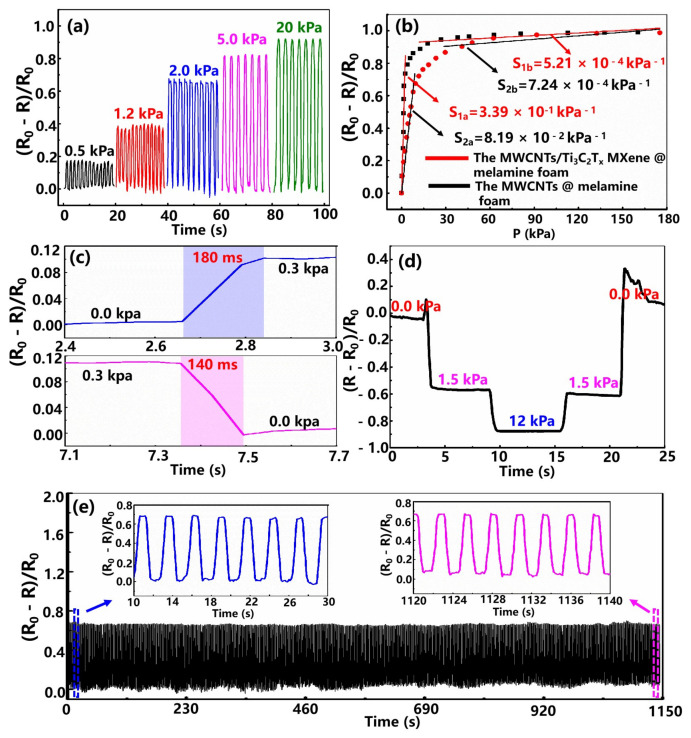
Performance characterization of the fabricated flexible pressure sensors. (**a**) Relative resistance variation of the MWCNTs/${\mathrm{Ti}}_{3}C_{2}T_{x}$ MXene @ melamine foam under serial pressures below 25 kPa. (**b**) Relative resistance change vs. the applied pressure of the different types of foam-shaped sensors. (**c**) The response/recovery time of the fabricated foam-shaped device is under 0.3 kPa. (**d**) The relative resistance change of the fabricated foam-shaped composite sensor under step-applied pressure. (**e**) Durability performance of the fabricated device under the pressure of 2 kPa at a frequency of 0.4 Hz.

With the assistance of our homemade test system, which consists of a data acquisition instrument, a force gauge and sport propulsion equipment, we systematically studied the related pressure-sensing properties of the fabricated pressure sensor. Figure 2a exhibits the relative resistance variation of the fabricated MWCNTs/${\mathrm{Ti}}_{3}C_{2}T_{x}$ MXene @ melamine foam under serial pressures below 25 kPa. As the loading pressure gradually increases, the resistance of the composite foam decreases correspondingly, indicating that the MWCNTs/${\mathrm{Ti}}_{3}C_{2}T_{x}$ MXene @ melamine foam can behave as an excellent pressure sensor.

As an extremely valuable parameter index for evaluating the performance of pressure sensors, the sensitivity is defined as $S=(\Delta R/R_{0})/\Delta P$, where ΔR, $R_{0}$ and ΔP represent the amount of change in resistance, the initial resistance and the applied pressure change, respectively. The relative resistance plots $\left(\Delta R/R_{0}\right)$ versus applied pressure for the different types of foam-shaped sensors are shown in Figure 2. It can be seen that the conductive pathways between adjacent porous skeletons gradually increases as the loading pressure increases, which exhibits two linear regions with different sensitivity values: a higher value in the low-pressure range and a lower value for higher pressures.

Between the two linear regions, the sensitivity varies significantly nonlinearly. The MWCNTs/${\mathrm{Ti}}_{3}C_{2}T_{x}$ MXene @ melamine foam exhibits a high sensitivity of 0.339 kPa${}^{-1}$ below 2.5 kPa and a wide operating range of up to 180 kPa, while the sensitivity of the MWCNTs @ melamine foam in the whole low-pressure range is significantly reduced to 0.0819 kPa${}^{-1}$. According to the above phenomena, the high sensing performance of the MWCNTs/${\mathrm{Ti}}_{3}C_{2}T_{x}$ MXene @ melamine foam sensor is mainly attributed to the synergistic effect of the internally connected MWCNT percolation network and the ${\mathrm{Ti}}_{3}C_{2}T_{x}$ MXene nanosheets adsorbed on the network.

The introduction of ${\mathrm{Ti}}_{3}C_{2}T_{x}$ MXene will make the multi-walled carbon nanotubes no longer agglomerate, and the MXene nanosheets between the layers are connected with the multi-walled carbon nanotubes to form a scaffold structure, which endows the sponge with good compressive properties. In addition, the presence of multi-walled carbon nanotubes can effectively prevent the stacking of MXene nanosheets, enabling the nanosheets to effectively slip when pressure is applied.

We also measured another critical parameter of the fabricated foam-shaped sensor: the response time. The obtained sensor exhibits response and recovery times of 180 and 140 ms under 0.3 kPa (Figure 2c), respectively, sufficient for low-frequency human motion monitoring. Figure 2d shows the relative resistance change of the fabricated foamed composite sensor under stepped-applied pressure. By using the self-made test system, the pressures of 1.5 and 12 kPa were gradually implemented on the sensor and held for a while, respectively, then the pressure was released to 1.5 kPa, and finally, the pressure was released completely.

It can be seen that, during the loading and unloading processes under the same pressure, the electrical output signals of the device are basically consistent, indicating that the device maintains high stability after the cycle process. The presence of the glitch is attributed to the resulting unstable pressure signal when applying and releasing pressure. In the actual application process of the sensor, the stability under multiple cycle tests is the basic requirement of the device.

The fabricated foam-like composite sensor was repeatedly loaded and unloaded at a frequency of 0.4 Hz under a pressure of 2 kPa, and the resistance response was also recorded as shown in Figure 2e. It can be observed that the magnitude of the resistance response is almost uniform after each loading–unloading cycle, and the resistance amplitudes are almost uniform at each loading–unloading cycle, indicating that the device features excellent long-term cyclic stability.

**Figure 3:**
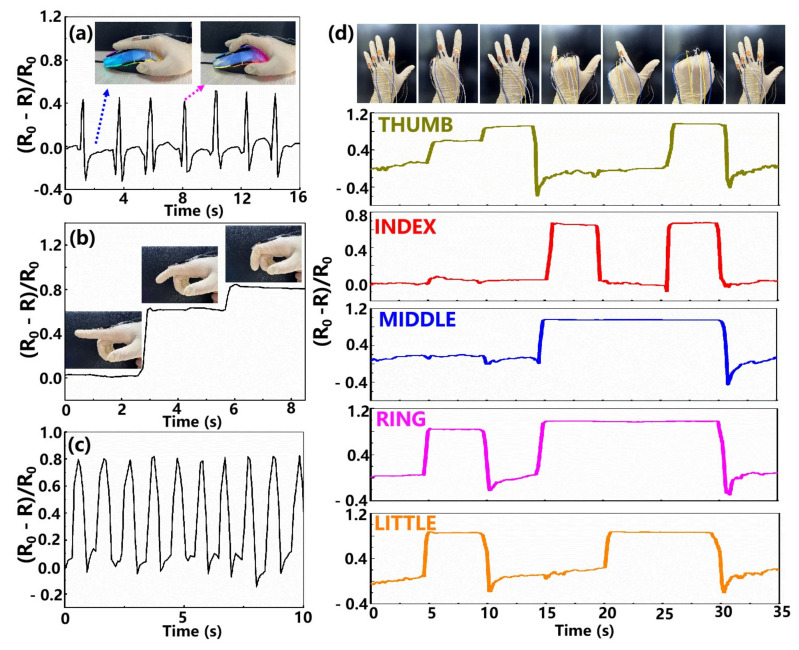
Monitoring of human gesture movements by the developed foam-shaped composite sensor. (**a**) The response signal of the obtained device in scrolling the mouse. (**b**,**c**) Detecting resistance change with an index finger (**b**) at various bending states, (**c**) and under repeated bending at the same angle. (**d**) The real-time response of the foam-like device to a sequence of Arabic numeral gestures.

Based on the excellent sensing properties, the fabricated foam-shaped sensor was used with success in human gesture monitoring. The foam-shaped composite sensor was first affixed to the joints of the index finger with the assistance of medical tape to convert physical deformation into the resistance signals. Then, the output signals of the fabricated sensor were recorded by the Keithley DAQ6510 data acquisition system under the state of scrolling the mouse and bending the finger as shown in Figure 3a–c. The peak-shaped response signal appeared when scrolling the mouse or quickly bending the finger (Figure 3a,c).

Moreover, when the same gesture action is repeated many times, the response signals show consistent characteristic peaks and valleys, indicating the excellent reliability of the sensor for detecting human gestures. Figure 3b illustrates that the resistance value of the device gradually decreases with the increase of the motion bending degree, which can be explained by the different degrees of compression of the sensor. The above result demonstrates that the fabricated sensor can be used to distinguish different states of finger bending.

The application capability of the MWCNTs/${\mathrm{Ti}}_{3}C_{2}T_{x}$ MXene @ melamine foam sensor for gesture detection is further demonstrated as shown in Figure 3d. Five independent MWCNTs/${\mathrm{Ti}}_{3}C_{2}T_{x}$ MXene @ melamine foam sensors were taped on the second knuckle of each finger of a glove to assess the signal of finger flexion and extension states. In this test, a series of Arabic numeral gestures were posed separately, and the interval between each gesture was 5 s. During the first 5 s, all fingers are extending, resulting in essentially no change in the resistance value of the fabricated sensor.

Immediately afterwards, the thumb, ring finger and little finger are bent to form the Arabic numeral “2”, giving rise to a significant decrease in the resistance value of the corresponding sensors. Similarly, when the Arabic numerals “4”, “6”, “8” and “0” are posed in sequence with the fingers, the resistance of the sensors on the curled-up fingers decreases correspondingly. When the Arabic numeral “5” is posed again, the resistance values of all sensors return to the initial state, which further proves the potential of the fabricated sensor in the field of gesture monitoring.

**Figure 4:**
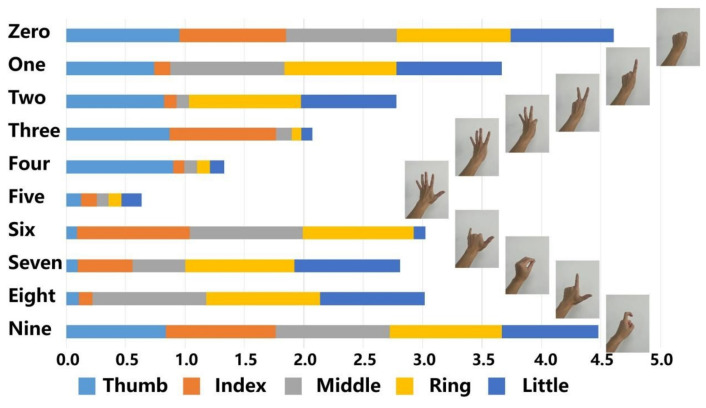
The average measurements of the gestures.

Subsequently, we investigated the feasibility of the fabricated foam-shaped composite sensor for gesture recognition by combing it with the algorithm. Gesture data of two subjects were collected using our prepared sensor. The subjects were two 25-to-30-year-old Asian men with normal hand movement functions, wearing the aforementioned sensor gloves and making gestures of Arabic numerals “0” to “9”. The gestures are shown in Figure 4, and the normalized resistance change rates $\left(R_{0}-R\right)/R_{0}$ corresponding to each sensor are recorded as the measurement results.

The relative resistance change was chosen instead of resistance to minimize the influence of the inconsistency of the initial resistances of the sensors on the measurement results because different lengths and curvatures of the fingers will produce different initial resistance values. In the experiment, each subject performed each gesture 30 times. The average value of the measurement results is shown in Figure 4 that the gestures of the numbers are of quite different resistance distributions, except for “0” and “9”. The gesture “0” has a slightly larger amplitude than “9”, likely because the fingers bend slightly more when representing “0”.

Hence, machine-learning methods can be used to recognize the gestures, such as decision trees based on statistical thresholds [29] or the support vector machine [30] based on the geometric distance between clusters. This paper used a decision tree with low computational complexity as the classifier for multi-classification tasks with small sample datasets, considering that the task is oriented towards wearable devices and possibly deployed to microcontroller units or edge computing units.

The decision tree divides the dataset according to the different judgment criteria of non-leaf nodes. The training algorithms can be roughly divided into two categories: one is based on information entropy, such as ID3 and C4.5 decision tree algorithms; the other is based on the Gini index, such as the CART (classification and regression tree) algorithm. The C4.5 [31] decision tree algorithm is used in our experiment for the classification task whose features are continuous values, discretizing the continuous values with binarization. That is, for a continuous feature, there is at least one threshold that can divide the sample space into two different classes, and the binarized feature can be represented as two branches of a root node.

**Figure 5:**
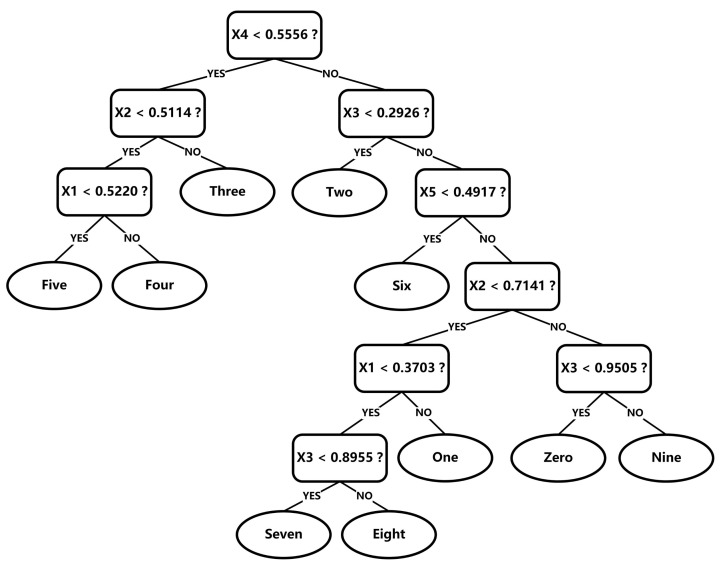
The binary decision tree model used for the gestures classification task, where X1–5 represents the normalized resistance change rate at the thumb, index finger, middle finger, ring finger and little finger, respectively.

If there are multiple thresholds, one can select the threshold that maximizes the information gain. The dataset is randomly divided into a 70% (420 samples) training set, a 15% (90 samples) validation set and a 15% (90 samples) test set. The decision tree model in Figure 5 is obtained through training. The platform that we used to realize the decision tree model is Scikit-learn [32] on Python 3.6. The decision tree has 10 leaves corresponding to 10 different gesture classes, which is the minimum number of leaves to achieve effective classification, and thus there is no risk of overfitting.

**Figure 6:**
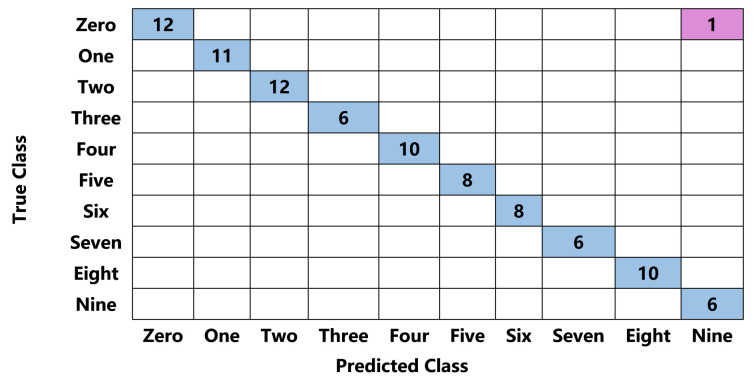
The confusion matrix of the test set. The color blue indicates the inference is correct while the pink indicates the inference is wrong.

The Confusion matrix of the decision tree on the training set is shown in Figure 6, where the predicted class is the prediction result from classifying a test sample through the decision tree model. The predicted class is compared with the true class to calculate classification accuracy. The average accuracy of the trained decision tree classification model on the test set is 98.9%. We then separately counted the number of correctly identified (predicted class matches the true class) and incorrectly identified (otherwise) samples in the test set.

Almost all gestures are recognized correctly, except that one “9” is wrongly identified as “0”. The sensed measurements of gestures of “0” and “9” are pretty similar, so they are easily misjudged during recognition. However, the classifier misjudged only one gesture of “0” as “9” in 13 tests. Hence, the above results clearly show that combing the fabricated high-performance foam-shaped composite sensor with the decision tree algorithm can unambiguously identify different gesture motions, demonstrating the remarkable application potential of our sensing device in the field of gesture recognition.

In addition, the sensor is designed for wearable devices, and thus the highest priority to be considered is the computational complexity of forwarding inference. The decision tree classifier is suitable for wearable device deployment because it can be implemented with only If-else statements, which are of low computational complexity. Therefore, a low-computing-power and low-cost micro controller unit (MCU) is sufficient to handle the classification calculation, such as an M0-core ARM MCU.

For comparison, we implemented another classifier based on a one-hidden-layer multilayer perceptron, a type of neural network. The model has five nodes in the input layer, eight nodes in the hidden layer and 10 nodes in the output layer. The activation function of the output layer is Softmax and of the other layers is Sigmoid. 80% of the dataset is used as a training set and 20% is used as a test set. After training, the accuracy of the network model on the test set is 98.3%, which is not greatly different from the decision tree method. However, even if a lookup table is used to calculate the activation function to save computing power, the model still requires about 120 multiplications, which is much more computationally complex than a decision tree classifier.

When using our proposed sensor for gesture recognition, only five input features from the five sensors are required, and the method can be applied in any environment, regardless of the shooting angle and lighting conditions, such as in darkness, flickering lights, or complex backgrounds. However, gesture images, such as the Jochen Triesch Static Hand Posture Database [33], require a fixed angle between the camera and the hand (usually a front view) to capture clear and complete gestures, which limits the user’s range of activities and will also affect the comfort of use.

In the presence of background objects, the classification accuracy drops from 94.3% to 86.2%. Moreover, the image needs to be cleaned after shooting to remove the background objects in the complex environment or to adjust the brightness before being classified. After that, a wavelet transform with complex Gabor-based kernels needs to be used to convert the 128×128-pixel images into graphs with 35 nodes and 72 edges with geometrical information. Classification is then achieved by matching images with geometric information. The computational complexity of transformation and classification is about $O(n^{2})$.

Even a small 8-bit grayscale gesture image has 128×128 input features and requires a huge amount of computation. Though the gesture images have a high degree of classification, it also has more input features, resulting in a greater amount of computation. Considering the computational complexity and usage environment, camera-based gesture images are not as suitable as our sensors-based method for wearable devices, such as VR gloves and electronic skin.

## 4. Conclusions

In summary, we designed and manufactured a flexible pressure sensor based on the MWCNTs/${\mathrm{Ti}}_{3}C_{2}T_{x}$ MXene @ melamine foam for human gesture monitoring and recognition by a simple dip-coating method. Ascribing to the synergistic effect of the internally connected MWCNT percolation network and the ${\mathrm{Ti}}_{3}C_{2}T_{x}$ MXene nanosheets adsorbed on the network, the obtained foam-shaped composite sensor exhibited extremely high sensing performance: high compression sensitivity (0.339 kPa${}^{-1}$), a desirable response time (<180 ms), a wide working range (up to 160 kPa) and excellent stability.

The practical applications of the fabricated foam-shaped sensor in distinguishing different states of fingers, such as scrolling the mouse, bending the finger and changing different gestures, were successfully demonstrated. Furthermore, the decision tree algorithm was utilized to recognize ten typical Arabic numeral gestures. The experimental results confirmed that the developed sensor combined with the machine-learning method can unambiguously classify different Arabic numeral gestures with an average recognition accuracy of 98.9%. Evidently, our fabricated foam-shaped sensor provides a promising strategy for gesture recognition tasks in exercise and rehabilitation training.

## Data Availability

Not applicable.
